# Translation and cultural adaptation of the HELPS Reading Fluency Program into Brazilian Portuguese: A report of systematic adaptation processes and initial evidence of efficacy

**DOI:** 10.3389/fpsyg.2023.1034749

**Published:** 2023-02-09

**Authors:** Maíra Anelli Martins, John C. Begeny, Simone Aparecida Capellini

**Affiliations:** ^1^Investigation Learning Disabilities Laboratory, Department of Speech and Hearing Sciences, São Paulo State University “Júlio de Mesquita Filho” (UNESP), Marília, Brazil; ^2^Department of Psychology, College of Humanities and Social Science, North Carolina State University, Raleigh, NC, United States

**Keywords:** reading fluency, intervention, reading development, automaticity, visual word recognition, HELPS program, language adaptation

## Abstract

**Introduction:**

Across multiples languages, research demonstrates the important relationship between reading fluency and comprehension. Put simply, a fluent reader has greater attention and memory resources to use higher-order functions in reading, resulting in better comprehension of text. Some reading fluency interventions have shown positive results in improving students’ text reading fluency and comprehension; however, this research has predominantly been conducted with English-speaking students. For instance, until this report, a comprehensive search revealed only one prior study that evaluated an intervention strategy designed to improve students’ reading fluency in Brazilian Portuguese and no prior studies evaluated an intervention *program* with that population of students.

**Methods:**

The main goals of this two-part project were to (a) systematically translate, culturally adapt, and pilot test the Helping Early Literacy with Practice Strategies (HELPS) reading fluency program for use in Brazilian Portuguese (referred to as, *HELPS-PB*); and (b) conduct a preliminary quasi-experimental study of the HELPS-PB program with 23 students in grades 3 to 5 who needed a reading fluency intervention.

**Results and Discussion:**

This report documents the processes and successful adaptation of existing English- and Spanish-versions of HELPS into a new HELPS-PB program. It also offers preliminary evidence showing that students receiving HELPS-PB significantly improved their text reading fluency comparted to students in a control group. Implications for research, practice, and the adaptation of reading fluency programs into other languages are discussed.

## Introduction

Of the various key components that are activated in the reading process (e.g., decoding words, understanding vocabulary, comprehending words on the page), reading fluency is equally important and is a multidimensional concept involving reading rate, accuracy, and prosody (Puliezi and Maluf, 2014; Hudson et al., 2020).

One of the earliest models used to explain the importance of reading fluency was proposed by LaBerge and Samuels (1974) and is called the information processing model. With exposure to the visual code (letters, spelling patterns, frequent words, and subsequent practice), the sets of letters of a word come to be recognized as a single unit, making the process increasingly automatic. Thus, attention resources for the visual decoding processes decrease, allowing focus to shift to other areas, such as the semantics (meanings) of the text being read and critical thought or analysis of the text.

As the reader acquires and improves text reading fluency skills, this frees attention and memory resources for the use of higher-order functions in reading, resulting in better comprehension (Laberge and Samuels, 1974; Hudson et al., 2020). Higher-order functions are related to cognitive abilities necessary for strong comprehension. For example, a reader must integrate the meaning of words and phrases into a meaningful whole, make inferences, monitor one’s own comprehension of text, and seek to build a coherent representation of the text in memory in order to integrate it with previous knowledge (Rapp et al., 2007; Oakhill et al., 2017; Pacheco and Santos, 2017).

Despite the advancement of research on instruction in reading (e.g., research on how to develop students’ fluency and improve reading comprehension), a large percentage of students around the world continue to struggle with basic reading proficiency. The Programme for International Student Assessment (PISA) represents just one well-known source for this global crisis in literacy development (OECD, 2019b). Reading difficulties affect students of all ages and demographic characteristics, but those who grow up with economic disadvantages are particularly at risk for significant difficulties in reading (e.g., OECD, 2019b; Soares and Bergmann, 2020; National Center for Education Statistics, 2022). Although a widespread lack of reading proficiency calls for global action, we next highlight some data and information about literacy in Brazil because this is the geographic location of the student participants discussed later in this paper.

### Reading proficiency in Brazil

The Brazilian National Common Curriculum Base states that literacy is a priority in the first two grades of elementary school so children can learn “the alphabetic writing system in an articulated way [along] with the development of other reading and writing skills [while involved] in diversified literacy practices” (Brasil, 2018, p. 59). However, multiple sources of evidence suggest that many students in Brazil do not develop proficient reading skills.

For example, one recent UNESCO report highlighted significant gaps in Brazilian students’ reading proficiency, showing, for instance, that only 3.6% of public-school students in Brazil complete elementary school with advanced reading skills (UNESCO, 2017). Additionally, data analyzed from PISA within Brazil—which included 597 public and private schools and 10,961 students—indicate that 50% of Brazilian students aged 15 years old had low reading proficiency (Araújo and Andriola, 2019; OECD, 2019a). This percentage is also highly consistent with national assessments of literacy in Brazil that included primary grade students (Brasil, 2013, 2021; Soares and Bergmann, 2020).

Overall, assessments of students’ reading performance in Brazil (Brasil, 2013, 2021), as well as international assessments (e.g., OECD, 2019a), suggest a critical need to improve students’ reading. This is extremely important in the early grades because it appears that most students identified as having reading difficulties continue to have them all the way into secondary school (Brasil, 2013, 2021; UNESCO, 2017).

### Reading fluency and related research in Brazil

Although reading proficiency involves developing a handful of essential foundational skills (e.g., phonemic awareness, vocabulary, and comprehension), reading fluency is one of those essential skills (Hudson et al., 2020; Rupley et al., 2020; Meggiato et al., 2021; Silvano and Godoy, 2022). Reading fluency is often defined as the ability to read aloud quickly, accurately, and with proper expression (e.g., Rasinski, 2006; Kuhn et al., 2010; Pinto and Navas, 2011). As we discuss in greater detail later, students’ development of reading fluency involves using evidence-based practice and motivational strategies, including strategies such as having students repeatedly read ability-appropriate text for a prescribed frequency and duration, having a proficient reader model fluent reading for a student developing fluency, using systematic error-correction procedures with words a student reads aloud incorrectly, and integrating motivational strategies such as goal-setting and structured praise (e.g., Therrien, 2004; Morgan and Sideridis, 2006; Lee and Yoon, 2017; Stevens et al., 2017; Padeliadu et al., 2021).

Given the importance of text reading fluency as an essential foundational literacy skill, as well as existing research validating a small number of intervention programs that improve students’ fluency, there is a critical need to utilize evidence-based intervention for the millions of Brazilian students who have not yet developed reading fluency (Puliezi and Maluf, 2014; Meggiato et al., 2021; Silvano and Godoy, 2022). To date, and after a comprehensive search for relevant literature, we identified only one existing study designed to evaluate intervention strategies to support Brazilian students’ reading fluency.

In that study, Pinto and Navas (2011) used fluency-based instructional strategies in an effort to improve fourth-grade students’ reading rate. During the five instructional sessions with each student (15 min per session), they used silent reading, modeling, and repeated reading strategies, as well as a prosody-based strategy in the first session. The results showed small but statistically significant improvements from pre- to-post-test in students’ reading prosody and error-rate, whereas the small growth in students’ number of words read correctly per minute (reading rate) was not statistically significant. In what appears to be the very first study designed to evaluate and improve Brazilian students’ text reading fluency, this study was important in emphasizing the need to strengthen students’ fluency and it offered an initial evaluation of a few basic instructional strategies that studies outside of Brazil have shown to be effective (Lee and Yoon, 2017). However, this study was also limited in several important ways. For example, applying only a few fluency-based instructional strategies in a more “basic” manner is unlikely to support students as much as using several evidence-based strategies that are implemented in the most empirically supported ways (Therrien, 2004; Morgan and Sideridis, 2006; Begeny, 2009; Stevens et al., 2017). Similarly, evaluations of comprehensive instructional *programs* (e.g., programs that provide all needed implementation materials, training, guidance, etc. that educators can access outside of a journal article) have important implications for usability and feasibility of such interventions outside of a research context. Methodologically, the study also had some important limitations, such as no inclusion of a control group and only involving students who did not appear to have reading difficulties.

### Purpose of this two-part study

Our discussion thus far emphasizes two key ideas. First, there is critical need to support students’ reading development, including development of fluency as a foundational skill. This fact is true in Brazil and in most countries around the world (e.g., Therrien, 2004; Pinto and Navas, 2011; Lee and Yoon, 2017; OECD, 2019b; Brasil, 2021). Second, there is a substantial gap in programming and research around reading fluency for students learning to read in Brazilian Portuguese. These two main facts served as the impetuses for this 5-year project that involved two main studies.

Study 1 sought to systematically translate, culturally adapt, and pilot test an existing reading program that (a) has the target goal of improving students’ reading fluency and confidence as readers, (b) has more than a decade of research supporting its effectiveness on students’ reading fluency and comprehension, (c) has been used with students in more than 60 countries, and (d) was available in English in Spanish at the beginning of this project. Specifically, we sought to systematically adapt and develop a Brazilian Portuguese version of the Helping Early Literacy with Practice Strategies (HELPS) program—which was originally developed in English (Begeny, 2009) and later adapted into Spanish, with the name of *Leamos para Avanzar* (Begeny, 2012). Systematic adaptation and development work for the Brazilian Portuguese version of HELPS also required translation, adaptation, and pilot testing of the reading passages (i.e., the HELPS curriculum of passages) that accompany the intervention program in English (Begeny et al., 2009) and Spanish (Begeny et al., 2012a). Collectively, Study 1 sought to document the systematic process of creating the Brazilian Portuguese version of HELPS (i.e., HELPS-PB), including documentation of the necessary pilot data needed to appropriately adapt and sequence the HELPS-PB curriculum of passages and related implementation materials. Study 1 sought to answer the following two questions: (a) will our empirically and theoretically based approach to translation and adaptation lead to successful development of HELPS-PB (as defined by data collected throughout the process and implementation observations occurring during pilot implementation) and (b) what key aspects of our development process were learned that may influence similar development processes in future work?

Study 2 was designed to build upon the development work from Study 1 by conducting an initial quasi-experimental study of HELPS-PB with students in grades 3 to 5 who lacked proficient reading fluency and needed a targeted reading fluency intervention. As a preliminary evaluation of the efficacy of HELPS-PB and the first known study to evaluate a reading fluency intervention program with students learning to read in Brazilian Portuguese, Study 2 sought to answer one main research question: do participants who receive HELPS-PB significantly outperform wait-list control group participants in text reading fluency, as measured by a standardized reading fluency assessment?

This research project was submitted to the Research Ethics Committee of the School of Philosophy and Sciences-CEP/FFC/UNESP-Marília-SP and approved under number 1.299.842, CAAE 50201915.9.0000.5406. The project approved by this CEP refers to all stages of the work, including Study 1 and Study 2.

## Study 1: Cross-cultural translation and adaptation of HELPS materials into Brazilian Portuguese

### Overview and context

HELPS is a structured, evidence-based program designed to improve students’ oral reading fluency (ORF) and confidence in reading. Several published studies (e.g., Begeny et al., 2010, 2011; Malouf et al., 2014; Mitchell and Begeny, 2014; Vess et al., 2018) and more than 10 consecutive years of comprehensive program evaluations have evidenced the effectiveness of HELPS in improving reading fluency and/or comprehension for a broad and diverse group of students, including but not limited to students in elementary and middle school, students for whom English is or is not the student’s first language, students with and without disabilities, and students who live in economically disadvantaged households. Based on several meta-analyses and systematic reviews of the available research on interventions designed to improve reading fluency and (as a result) reading comprehension (e.g., Chard et al., 2002; Therrien, 2004; Morgan and Sideridis, 2006; Lee and Yoon, 2017), HELPS includes each of the known evidence-based strategies for building reading fluency. This includes strategies such as repeated reading, systematic error correction, model reading, performance feedback, goal setting, and structured motivation systems (Begeny, 2009). HELPS instructional sessions last approximately 15–20 min, it is recommended that students receive at least three sessions per week for at least 30–50 sessions, the program can be implemented effectively in a one-on-one or small group context, and HELPS can be used effectively in-person or virtually (Begeny, 2009, 2018b; Vess et al., 2018; Richardson, 2019; Musti-Rao et al., in press).

In an effort to promote educational equity and a more just society, all HELPS program implementation and training materials are made available for free by the program’s lead developer and are disseminated by Helps Education Fund, a United Stated 501(c)(3) non-profit organization that provides more than a dozen research-validated programs and services for free or low cost. As part of this work, efforts are made to work with educators, researchers, and program developers around the globe who have interest in translating and adapting any of Helps Education Fund’s programs and materials into additional languages. Consistent with all other programs and services offered by Helps Education Fund, any newly adapted or translated Helps Education Fund programs, such as HELPS, must (a) be comprehensively developed, (b) evidence some level of effectiveness with the intended beneficiaries (e.g., students), and (c) be made available from Helps Education Fund for free or low cost.

This overall context served as the foundation for the collaborative partnership that sought to facilitate Study 1 of this report. To conduct Study 1, a translation and adaptation license agreement was requested and granted by the lead developer of HELPS. Directed by the first author of this report, Study 1 involved approximately 4 years of collaborative development and pilot-testing work before Study 2 of this report could be initiated.

Finally, because the goal of Study 1 was to complete a translation and adaptation of HELPS materials into Brazilian Portuguese, it is important to highlight that the appropriateness of reading intervention programs that are applied and adapted to different languages can be influenced by the orthographic transparency of each alphabetic language system. As described in detail by others (e.g., Cardoso-Martins and Navas, 2016; Borleffs et al., 2017), Portuguese is at an intermediate level of orthographic transparency compared to English (which is more opaque) and Spanish (which is more consistent). The more that grapheme and phoneme correspondences are consistent for the learner, the better they will be able to learn decoding skills at the beginning stages of literacy development. As such, reading difficulties for Brazilian students that occur during or after grade 1 or 2 have a relatively high likelihood of being influenced by fluency difficulties, due to the relatively consistent or “transparent” nature of Portuguese (Borleffs et al., 2019). In fact, research with Brazilian students confirms this idea, with evidence suggesting that difficulties in reading fluency play an important role in reading comprehension from the beginning of learning to read, such as for grade 1 students (Cardoso-Martins and Navas, 2016).

### Method

The cultural adaptation of the HELPS program included the translation of (a) a comprehensive instructor’s manual (Begeny, 2009), which included 152 pages of all the needed implementation materials, answers to frequently asked implementation questions, a brief summary of relevant research and context for using HELPS, as well as overall guidance for teachers on how to most effectively use the program; and (b) a curriculum of 100 passages (narrative and expository text) for the students to read as part of program implementation. Henceforth we will simply refer to these two documents as the “instructor’s manual” and “curriculum.” In addition to the translation and back-translation of these materials, the curriculum passages were adapted for cultural fit and student data were systematically collected to level the passages of the curriculum for Brazilian students, adapt the HELPS goal-setting procedure according to norms for students to read in Brazilian Portuguese, and adapt the HELPS program’s Placement Assessment that specifies where a student should start in the curriculum of passages.

At its foundation, the methodology for translation and adaptation of this program was based on methods described by Cassepp-Borges et al. (2010) and Alexandre and Coluci (2011), as well as studies that used these authors’ techniques (e.g., Manzi-Oliveira et al., 2011; Constant et al., 2014; Holst et al., 2016; Brito and Faro, 2017). For example, we followed guidance from Cassepp-Borges et al. (2010), who presented techniques for adapting psychological instruments from one culture to another in ways that aim to reduce cultural bias. Based on work by Alexandre and Coluci (2011) regarding standardized, international guidelines to help ensure the quality of adapted materials, we utilized steps proposed by these authors that were described as essential for this type of work: initial translation, synthesis, translation back to the original language, review by a committee, and performance of a pre-test (pilot study).

**Figure 1:**
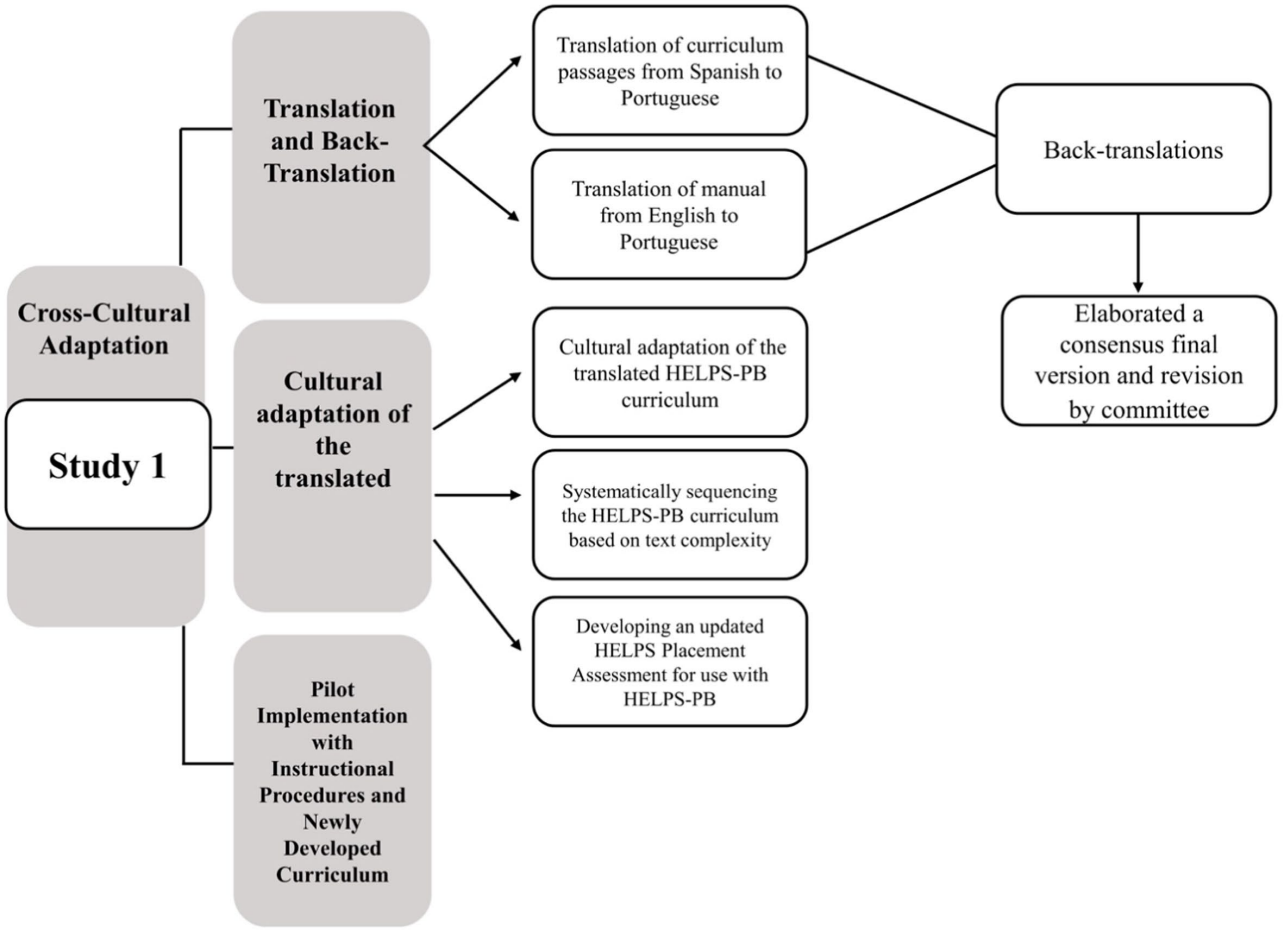
Diagram for the primary stages and activities of Study 1.

We also used geographic and localized contexts, based on conceptual and practical models of internationalization in psychology and education (e.g., Arfken, 2012; Begeny, 2018a, 2019; Begeny et al., 2021). Finally, adaptation procedures involved collaborative work among all authors of this paper to account for specific program-related guidelines that are unique to using and developing the HELPS program. What follows is a summary of the primary steps we used for Study 1: (a) translation of HELPS materials, (b) cultural adaptation of the HELPS curriculum, (c) systematically sequencing the HELPS-PB curriculum of passages based on text complexity, (d) developing an updated HELPS Placement Assessment for specific use with HELPS-PB, and (e) pilot testing of the newly developed HELPS-PB program. Figure 1 also presents a visual depiction of the primary stages and activities of Study 1.

#### Translation and back-translation of the HELPS manual and curriculum of passages

To prepare for translation, the Spanish version of the HELPS curriculum of passages (Begeny et al., 2012a) and the English version of the HELPS instructor’s manual (Begeny, 2009) were selected as the two key sources of text to translate. These were selected because a translation of the HELPS curriculum from Spanish to Brazilian Portuguese (both Latin languages) would likely be easier compared to translating the English version of the curriculum to Brazilian Portuguese. However, the Spanish version of the HELPS Instructor’s manual is not a fully comprehensive version (e.g., it excludes summaries of relevant research about the instructional strategies used in HELPS), so the English (and fully comprehensive) instructor’s manual was used for translation. For concision, unless otherwise stated, we will subsequently use *Portuguese* to refer to *Brazilian Portuguese,* though we acknowledge that written and spoken Portuguese outside of Brazil (e.g., in Portugal) is sometimes different.

The choice to translate the HELPS curriculum from Spanish to Portuguese was also done because of Latin American cultural constructs and relative similarities. For example, there are corresponding elements between the Brazilian and the Castilian cultures, which facilitates translation because it decreases the probability of idiomatic and grammatical incompatibilities (Cassepp-Borges et al., 2010). The person selected to translate the HELPS curriculum was a native Portuguese speaker who is also fluent in Spanish. The person selected to translate the HELPS manual was a native Portuguese speaker who is fluent in English. Following the instructions of Cassepp-Borges et al. (2010) and others (e.g., Herdman et al., 1998; Reichenheim and Moraes, 2007; Almeida et al., 2013) to ensure a valid and independent reverse translation, the translated versions of the manual and curriculum were translated back into the original language by professionals who did not participate in the first stage translations and did not know about the HELPS curriculum or manual.

To unify a preliminary version of the curriculum and manual, a committee then met to assist in the consolidation of the translations, minimizing possible linguistic, psychological, cultural, and comprehension biases found in the simple and reverse translations (Cassepp-Borges et al., 2010). The committee included one of the translators of the original in English into Portuguese (also fluent in the Spanish language), a researcher connected to this project, and a member external to this project but an expert in the area of pedagogy. In one meeting, all questions divergent from the original HELPS materials were analyzed, suggestions were discussed, and then modifications were made through the process of dynamic equivalence, including revisions to address any linguistic or conceptual issues.

Finally, the orthographic and grammatical revisions of all content was completed by two additional professionals who were highly qualified for this work: a retired teacher who taught school-aged students literacy and grammar for 30 years in Brazil, and a Portuguese language teacher with 47 years of experience revising the Portuguese language (through grammar and spelling reviews). The reviews were performed in sequence, that is, initially by one of these professionals and then independently reviewed by the second teacher. After all translation steps were completed and we had our initial HELPS-PB curriculum of passages, that curriculum was now ready for cultural adaptation.

#### Cultural adaptation of the translated HELPS-PB curriculum

The original HELPS curriculum was developed with the overall goal of creating a large set of reading passages that could be used effectively in English with students who are working to strengthen their text reading fluency—and in particular, the passages were developed for use with the HELPS program. The authors who developed the original HELPS curriculum in English (Begeny et al., 2009) considered more than a dozen passage characteristics and parameters that would be used in the curriculum development process. Chapter 3 of Begeny’s (2009) HELPS Manual summarizes the key considerations and characteristics of the HELPS curriculum, but examples of considerations included intentional creation of passages that: (a) have a complete story/passage in approximately 150–200 total words; (b) cover a variety of topics that would likely be of interest to a wide range of students, particularly primary school students; (c) incorporate themes and character names that collectively reflect cultural diversity and at least some global relevance; (d) offer both narrative and expository text, with the latter type of text being particularly age-appropriate for learners of all ages; and (e) across all 100 passages, collectively integrate 100% of the words from the Dolch High Frequency Word Lists (Dolch, 1948, 2007). The authors who translated and adapted the HELPS curriculum into Spanish (Begeny et al., 2012a) likewise attended to the considerations and parameters used in developing the original curriculum, as applicable for Spanish-language text.

With development of the HELPS-PB curriculum, these considerations were also used and therefore required intentional cultural adaptation of several passages. For example, the names used in passages and the themes of each passage were carefully examined by the curriculum developers to determine whether any passages should be modified or completely excluded from the HELPS-PB curriculum. This process included ongoing discussion about appropriate adaptations of the passages until there was full agreement among the HELPS-PB authors. After the curriculum adaptation process, and consistent with the development process for HELPS in English and Spanish, the HELPS-PB passages were ready to be systematically sequenced based on the text complexity of each passage—as measured by students’ ORF scores.

#### Systematically sequencing the HELPS-PB curriculum based on text complexity

The original HELPS curriculum was systematically sequenced from the least difficult to the most difficult passages. For this purpose, the developers used the mean oral reading rate (i.e., words read correctly per minute; WCPM) from hundreds of students in grades 1–4 to identify and sequence 100 usable passages that met the goals of curriculum development. More than 100 passages were originally written in the development process and standard deviations of students’ WCPM were used to exclude any passage that resulted in too much variability (i.e., any passage that was difficult for some readers and easy for other readers was not used because it reflected too much variability in text difficulty level). This overall approach was used for both practical and empirical reasons, and passage sequencing did not rely on applying readability formulas to each passage because there are still many criticisms and limitations of using such formulas to predict text that will be more or less difficult for a student to read with fluency or comprehension (for an extended discussion, see Begeny and Greene, 2014).

In considering the HELPS-PB curriculum, scholars suggest that English-language works translated into Portuguese tend to generate more complex texts than the original English version (e.g., Pasqualini et al., 2014). Considering this and the need to level the HELPS-PB passages from the least difficult to the most difficult for Brazilian students learning in Portuguese, it was therefore necessary to empirically assess the linguistic complexity of the adapted HELPS-PB passages by assessing Brazilian students’ oral reading rate and accuracy with each passage (i.e., their WCPM). Accordingly, a sample of students was selected and we engaged in a four-step process to determine the appropriate sequence of the HELPS-PB passages from least to most difficult, when read for purposes of fluency.

*Participants and overview of assessment procedures.* Prior to the beginning the passage sequencing process, parents or guardians of the participating students signed an informed consent form to authorize the study, which was in compliance with the resolutions of Brazil’s National Health Council CNS 466/12. In total, 72 third-grade students participated, 37 boys (51.4%) and 35 girls (48.6%), all students were 8 or 9 years old, *M* = 8.72, *SD* = 0.44. All students attended a public elementary school in the Midwest region of São Paulo. The participating school was the same for all stages of Study 1 and the same school participated in Study 2. The inclusion criteria for students to participate were (a) parental consent; (b) visual and auditory acuity within the normal range, as described in the school records and teachers’ reports; (c) no presence of a neurological, behavioral, or cognitive disorder.

The process of sequencing the HELPS-PB passages included the five main steps summarized below. When a step involved obtaining a student’s WCPM score, this was done by a trained assessor administering the standardized ORF assessment procedure (e.g., providing brief, specific directions for the student, timing the student’s reading for 1 min, and recording specified errors in reading). See Chapter 4 of Begeny (2009) for specific administration directions and scoring rules.

*Step 1*. A sub-sample of students and passages were selected to help us identify three HELPS-PB passages that are highly similar in difficulty level so that those three passages could later be used to identify our “homogenous-reader assessment pool” (i.e., a group of students from the 72 who have roughly the same level of ORF). To achieve this, we first selected HELPS-PB 10 passages. These 10 were the passages that Begeny (2009) reported as representing distinct levels of difficulty within the English version of the passages. There were two passages at each distinct level of difficulty, resulting in 10 total passages.

Although translation and adaptation of these passages into Portuguese undoubtedly changes the level of difficulty compared to the English-version passages, a goal of Step 1 in this process was simply to estimate roughly different difficulty levels of 10 total passages so that we could then identify (in Step 2) three passages that appear to be highly consistent in difficulty level. Also part of Step 1, we identified a reasonably sized sub-sample of students to read the 10 aforementioned passages. Specifically, of the 72 total participants, we had teachers nominate students who they reported as having grade-level reading skills (i.e., not with below average or advanced skills, but those with skills expected of third grade) and then randomly selected 12 of those students to participate in Step 2.

*Step 2*. The 12 aforementioned students read the 10 passages described in Step 1, and from that we averaged each student’s WCPM score for each passage. Of the 10 passages and based on average WCPM scores, we then identified three passages that showed roughly the same level of difficulty. These three passages served as the “screening passages” to determine (of the 72 total students) which of those students would be appropriate as participants in the *homogenous-reader assessment pool*.

*Step 3*. Of the three passages with roughly the same difficulty level identified in Step 2, we then administered those three passages to all 72 participants and each student’s median WCPM score represented their overall ORF score for the purpose of our sequencing process. Using the median WCPM score across three passage with similar difficulty level is well-substantiated and commonly used as best-practice in determining a student’s ORF. From this score, we identified 29 total students (15 female, 14 male, mean age = 8.51, SD = 0.49) who had highly similar levels of ORF [*M* = 64.75 WCPM; approximately the 35th percentile based on norms from Martins and Capellini, 2021] and this sample of participants served as our *homogenous-reader assessment pool.*

*Step 4*. Of the 29 students in the homogenous-reader assessment pool, each student read all 100 passages of the HELPS-PB curriculum over approximately a 2-week period. Each passage was printed on one A4 size sheet of paper and in a font size that was easy for students to read. The passages were presented in a binder-type folder so that each passage was presented in a uniformly straight manner and so that the student did not have access to the next passage before the reading began.

Data collection for this step involved the trained assessor administering 10 passages per day to individual students. The student always read each passage for only 1 min and the assessor obtained a WCPM score per passage. Consistent with the development of the original HELPS curriculum, a maximum of 10 passages were read daily (requiring approximately 12 total min for the day’s entire assessment session) so that students would be less likely to experience fatigue, inattention, or challenges with working memory—and thereby providing a context to obtain valid reading assessment data. Also, each assessment session took place at the student’s school, during the school day, and in a quiet room provided by the school principal.

The sequence of texts used in the assessment followed the sequential order of the passages (from 1 to 100) in the Leamos para Avanzar curriculum. The assessment process began with the same text for all children and followed with the same sequence of 10 passages presented per day to all children.

*Step 5*. Based on the sample of 29 students reading all 100 passages, we then calculated the mean and standard deviation of each passage. For the purposes of the HELPS instructional program and its ability to improve students’ fluency, passages in the curriculum should have relatively low variability. Thus, a passage (from the data collected in Step 4) was included within the final HELPS-PB curriculum if the passage showed reasonable variability across students’ performance (i.e., it was included if the standard deviation was less than 15.5 WCPM). Of the included passages (*N* = 95), these were sequenced from the highest to the lowest WCPM averages (with higher WCPM scores reflecting relatively easier passages) and HELPS-PB passages were sequenced accordingly. Table 1 shows the final sequence of each HELPS-PB passages as well as the respective WCPM and standard deviation. More details on the process of adapting the texts and their sequencing can be found in the Results of Study 1.

**Table 1:** Sequence of HELPS-PB passages based Study 1 WCPM averages.

Passage # for HELPS-PB curriculum	Average WCPM	Standard deviation	Passage # for HELPS-PB curriculum	Average WCPM	Standard deviation
Excluded	78.3	15.8	47	66.8	12.5
Excluded	77.5	15.6	48	66.8	10.0
1	77.2	11.7	Excluded	66.7	15.5
2	77.1	10.6	49	66.6	13.9
3	76.6	13.6	50	66.5	13.1
4	76.2	13.2	51	66.1	14.2
5	75.7	12.8	52	65.7	11.1
6	75.7	12.3	53	65.6	11.1
7	75.2	10.4	54	65.6	11.7
8	74.9	15.1	55	65.2	9.7
9	74.8	14.4	56	64.9	13.7
10	74.8	12.6	57	64.8	9.1
11	74.5	11.1	58	64.7	9.5
12	74.4	13.0	59	64.6	13.1
13	73.8	11.7	60	64.6	14.7
14	73.8	12.9	61	64.2	10.7
15	73.4	11.0	62	63.6	13.1
16	73.3	13.0	63	63.6	12.5
Excluded	73.3	15.5	64	63.4	13.7
17	72.9	13.4	65	63.1	14.4
18	72.4	14.4	66	62.3	13.9
19	72.1	11.7	67	62.0	13.2
20	71.8	13.5	68	61.8	13.7
21	71.7	11.2	69	61.7	11.4
22	71.3	10.4	70	60.8	8.6
23	71.1	9.9	71	60.7	10.5
24	70.2	11.0	72	59.2	11.7
25	70.1	10.3	73	59.0	10.8
26	70.0	14.7	74	58.7	10.9
27	70.0	14.0	75	58.6	12.8
28	69.7	12.0	76	58.5	10.3
29	69.5	15.2	77	57.9	10.8
30	69.4	12.9	78	56.4	12.4
31	69.2	11.7	79	56.2	9.9
32	69.1	12.6	80	55.6	10.7
33	69.1	13.3	81	55.4	11.8
34	69.0	11.8	82	55.2	7.8
35	68.8	11.3	83	55.0	13.2
Excluded	68.6	15.5	84	54.9	10.8
36	68.5	13.1	85	53.9	12.9
37	68.3	13.0	86	53.7	11.3
38	68.3	12.7	87	53.4	10.6
39	68.2	9.4	88	52.3	12.4
40	67.9	12.3	89	51.3	10.6
41	67.9	10.1	90	49.2	12.0
42	67.9	14.3	91	44.9	10.9
43	67.7	13.3	92	44.6	8.5
44	67.4	12.7	93	43.8	8.7
45	67.4	10.3	94	38.4	8.4
46	67.1	13.8	95	35.2	6.1

WCPM, words read correctly per minute.

#### Developing an updated HELPS placement assessment for use with HELPS-PB

According to the HELPS Instructor’s Manual, “the ideal starting point for a student in the HELPS curriculum is one in which the student will regularly meet his reading goal” (Begeny, 2009, p. 53). Research and program evaluations with HELPS also shows that an ideal starting point in the curriculum passages is the point at which the student reads a passage with approximately 20–30 WCPM less than the student’s specific Reading Goal and it is usually the case that a student’s WCPM score on each passage improves by approximately 20–30 words after 1–3 HELPS sessions (Begeny, 2009). With this, by starting a student in the curriculum at a passage where they will read approximately 20–30 WCPM less than the Reading Goal, this generally allows the student to regularly achieve the Reading Goal after 1–3 HELPS sessions and this logic is strategically designed to increase students’ reading fluency, motivation for the program, and reading confidence. However, when considering the large number of students who may receive HELPS, it is neither beneficial nor time-efficient for educators to have each student read every single passage in the curriculum to determine the optimal starting point per individual student. Rather, a data-based and time-efficient system must be in place to determine exactly where in the curriculum a student should begin once the HELPS instructional sessions commence.

That data-based system was developed for the English and Spanish versions of HELPS and is referred to as the HELPS Placement Assessment. More specifically, before a student begins receiving HELPS instructional sessions, a brief (usually 4–12 min) and structured assessment allows the educator to determine exactly what passage number in the curriculum a student should start with simply by having the student read a small number of pre-selected passages in the curriculum. Begeny (2009) describes the exact steps and rationale for the Placement Assessment, and the steps are likewise used for HELPS-PB.

It is beyond the scope of this paper to detail all the steps to administering the HELPS Placement Assessment, but because the HELPS-PB curriculum has its own sequence of passages (as shown in Table 1), it is necessary for this report to summarize how Study 1 completed the Placement Assessment decision-making rules for specific use with HELPS-PB. First, our decision-making rules followed the same logic and criteria used to develop the original HELPS Placement Assessment (e.g., the WCPM criterion table for the starting point in the curriculum was made with an interval of 20–30 words less than the student’s reading target, and this was calibrated appropriately for each grade level). Second, we needed to identify 10 appropriate passages that would represent each of the five “Levels” that are integrated in the Placement Assessment. The term *Level* is simply used to describe some Placement Assessment procedures and does not reflect the level of education, grade level, or the ability of the student.

Consistent with past procedures for developing the HELPS Placement Assessment, two passages should be selected for each of the five levels, with each level reflecting meaningfully different difficulty of the passages. For example, Level 1 passages are meaningfully easier than Level 2 passages; Level 2 passages are meaningfully easier than Level 3 passages, and so forth. Also, passages with lower standard deviations are best to select for the Placement Assessment procedures. Based on these and related rules for passage selection, the passages selected for the HELPS-PB placement assessment are as follows: Level 1 (passages 2 and 7); Level 2 (passages 23 and 25); Level 3 (passages 48 and 55); Level 4 (passages 61 and 70); and Level 5 (passages 79 and 82).

#### HELPS-PB pilot implementation with instructional procedures and newly developed curriculum

With each of the earlier procedures in Study 1 completed, this allowed us to then pilot HELPS-PB implementation with students. This pilot involved using both the newly translated HELPS-PB instructional procedures and materials (all available in the HELPS-PB Instructor’s Manual; Begeny et al., 2018a) as well as the now-finalized HELPS-PB curriculum of passages (Begeny et al., 2018b). This piloting of all HELPS-PB procedures and materials sought to verify whether the procedures (e.g., instructional steps, directions for students) used during the intervention are understandable by the target audience and to determine if students and teachers show responsiveness that is generally similar to what happens when HELPS is used in English or Spanish (based on past implementation of HELPS in these languages).

##### Participants

For the HELPS-PB pilot implementation, standard HELPS screening procedures (described next and within Begeny, 2009) were performed using Brazilian Portuguese ORF norms (see Martins and Capellini, 2021) to select students who could benefit from the HELPS-PB program. More specifically, to select the students who would participate in the pilot implementation, ORF scores were obtained from a sample of third to fifth grade students (*N* = 174) from one elementary school in the region of São Paulo. Prior to obtaining ORF scores, a parent or guardian of each student provided consent for participation. To participate, students also had visual and auditory acuity within the normal range and no presence of a neurological, behavioral, or cognitive disorder.

After administration of the ORF assessment with the 174 students (52 from third grade, 60 from fourth grade, and 62 from fifth grade), only students with reading difficulties were eligible to participate, as reflected by an ORF score between the 25th–50th percentile for the student’s respective grade level. Given the main purpose of this pilot (i.e., to understand whether the HELPS-PB procedures and materials were usable for students and instructors and whether sessions appeared to have the same general “feel” and benefits as HELPS when used in English or Spanish), we selected 6 students to participate (two each from the third, fourth, and fifth grades). Important to highlight, the participants involved with this pilot were not eligible to participate in the quasi-experimental study (i.e., Study 2 of this report, described later) and did not participate in any other stage of this study.

##### Materials and procedures

The newly translated and adapted HELPS-PB materials were used in the pilot. Intervention procedures followed each of the overall HELPS implementation steps that were originally developed (see Begeny, 2009), but all directions to students and corresponding materials were in Portuguese. For this pilot, six intervention sessions were conducted with each student and took place at each student’s school. Sessions were provided individually (interventionist and student only) and in a space provided by the school coordinator. Consistent with HELPS implementation recommendations, each student received three session per week. The duration of each session was approximately 15 min.

The lead researcher served as the interventionist during pilot implementation. In preparation for Study 1, she received the most intensive approach to HELPS training, which included 12 h of face-to-face training and structured practice activities that were facilitated by the program’s developer. At the beginning of training, a workshop was provided by the program’s developer to (a) address relevant instructional and theoretical questions, (b) teach workshop attendees how to implement the program, and (c) offer attendees structured practice opportunities with feedback. After additional practice, the lead researcher was eventually observed and verified by the program developer to be able to consistently implement the program with 100% fidelity.

To observe the overall usability of the program during the pilot implementation, we sought to gather information about some key questions. These questions were consistent with similar work of other researchers (e.g., Canhota, 2008) and guided by our interest in ensuring that HELPS-PB would be ready for Study 2. For example, we sought to understand: (a) the feasibility of implementing HELPS-PB three times per week in the participating school, and with sessions aimed to last approximately 15 min (which is important to assess because past research with HELPS had not taken place in Brazilian schools); (b) whether all HELPS-PB procedures and instructions were equally understood by all participating teachers and students; (c) whether students generally increased their WCPM on the passage practiced during each HELPS-PB session (which would be expected, based on past work with HELPS); and (d) whether students generally seemed motivated and engaged during each session.

### Study 1 results

Given the goals of Study 1, we sometimes integrated data or related information about “results” in the prior sections if doing that could enhance understanding and readability of this report. This section, however, summarizes some key results of Study 1 that have not yet been specified.

#### Translation and back-translation of the original versions of HELPS

There are three main results to report of the translation process. First, based on conversations among those involved with translating HELPS-PB, it was decided to keep the version of the American name of the Program: *“Helping Early Literacy with Practice Strategies* (HELPS)” and adding “Brazilian Portuguese (PB).” As such, the finalized program name was determined to be HELPS-PB. Second, unlike the various modifications and the use of the semantic equivalence process used in the translation of the Leamos para Avanzar curriculum passages to HELPS-PB passages (e.g., searching for another word that best describes the meaning in a sentence), the translation of the Instructor’s Manual from English into Portuguese typically did not require semantic equivalence processes because most of the language used in the manual is considered more technical-scientific. As such, translation of the manual allowed for a more rigid translation, referred to as word-word translation by Barbosa (2004). Third, from the back-translation and observations of the professionals trained by the translation committee, we observed that the translated part of the *HELPS* Manual was very similar to the retranslated or linguistically faithful material, demonstrating that this version closely approximated the original, maintaining a conceptual equivalence.

#### Adaptation of the HELPS-PB curriculum passages

It was possible that none of the themes of passages within the Leamos para Avanzar curriculum (Begeny et al., 2012a) would be analyzed in Study 1 as having content directly related to Brazilian culture. To discuss the possible need for exclusion and/or replacement of the passages, a meeting was held with the author of the original HELPS program, who encouraged and authorized replacement of any passages if that would help enhance the HELPS-PB curriculum’s themes to be more familiar and applicable to Brazilian students. When the Leamos para Avanzar curriculum was adapted from English into Spanish, three passages from the original HELPS program were excluded due to insufficient fit of culturally appropriate themes. Those three passages were replaced with three new passages that were developed to have themes and content closer to the reality of many students in Latin American. After analyses of passage content in the adaptation of HELPS-PB, it was decided to keep all the passages from the Leamos para Avanzar curriculum, and new themes generally seemed appropriate for Brazilian students.

During the adaptation analysis, words of foreign origin that had been incorporated into the vocabulary of the Portuguese language were retained within the curriculum. In a meeting with the committee, and in consultation with the Michaelis Modern Portuguese Language Dictionary (online version)^1^, it was decided to keep these “borrowed words,” called anglicisms, due to their common use. In fact, many of the words can already be found in Brazilian Portuguese dictionaries, are part of the Brazilian culture, and have been systematically incorporated into daily life of Brazilians. Some examples of words maintained in the translation process of the curriculum passages are: milkshake, pizza, video game, picnic, laser, kart, and guacamole.

Unlike Anglicisms, other foreign words were retained from the original translation even though they were not incorporated into Portuguese, as they describe words and foreign behaviors that were the subject of the passages and reflected cultural diversity of different countries. This included words such as *plátano banano, cambur, guineo, avocato, and oonch neech*. To mark the distinction of foreign words or expressions that are not included in the dictionary, they were written in italics within the HELPS-PB curriculum, thus highlighting them as foreign words. If the students did not read these words correctly, they were not considered errors.

#### HELPS-PB pilot implementation

In seeking to understand program usability of HELPS-PB during pilot implementation, we observed the following. First, the 15 min allotted for implementation was adequate to use all program steps with fidelity and it was likewise observed that students’ school routine allowed for three sessions per week. Classroom teachers knew when and how each student would be met by the interventionist to receive intervention and teachers reported feeling comfortable with the process.

Pilot implementation also revealed that all students, regardless of grade, easily understood the commands and instructions used during each HELPS-PB session. These observations suggested that although some of the fluency-based activities may have been somewhat unfamiliar with students’ typical school-day experience, they understood how to engage in the activities and showed no signs of disliking the activities. Rather, students showed and verbalized that they enjoyed the sessions, maintained age-appropriate engagement throughout each session, and liked the praise and systematic motivational system integrated within the program. Based on these observations, the program development team did not see a need to modify any of the procedures, directions for students, or methods for gathering reading data during each session.

The pilot also showed that students routinely increased their WCPM on passages from the beginning to end of the session, which is what would be expected and evidenced if the activities were, in fact, helping students improve their fluency on the passage practiced in each respective session. This was also important to find because the six students who participated in the pilot had started on different passages and each read multiple passages (range = 3–5) across the six pilot sessions. Additionally, the interventionist had no difficulty completing the student’s Progress Tracking Form or using the graph or motivational Star Chart.

## Study 2: Quasi-experimental evaluation of the *Helping Early Literacy with Practice Strategies* program in Brazilian Portuguese (HELPS-PB)

### Method

#### Participants

As described previously, the pilot implementation (conducted as part of Study 1 of this report) involved an ORF screening assessment with 174 students in grades 3–5. This process sought to identify students who could benefit from the HELPS-PB program as an intervention targeting students with difficulties in text reading fluency. Specifically, Martins and Capellini (2021) established ORF norms in Brazilian Portuguese and recommended that students in need of targeted fluency intervention are those students who fall within the 25th–50th percentile. Accordingly, Study 2 included students within that sample of 174 who (a) fell within the 25th–50th percentile range; (b) did not participate in the pilot implementation summarized in Study 1; (c) had visual and auditory acuity within the normal range; and (d) had no presence of a neurological, behavioral, or cognitive disorder. A total of 23 students met these inclusion criteria. Intervention staffing at the time of the study allowed for up to 15 students to receive approximately 30 sessions of HELPS-PB, so 15 students were randomly selected to receive HELPS-PB (i.e., experimental group students) and all remaining students (*n* = 8) were randomly assigned to a wait-list control condition.

Of the 15 students in the experimental group, five were in third grade, five in fourth grade, and five in fifth grade. Of the eight control group students, two were in third grade, four were in fourth grade, and two were in fifth grade. All control-group students received the HELPS-PB intervention after Study 2 was completed, as suggested in the National Code of Ethics in Human Research.

#### Materials and procedures

##### Assessment of oral reading fluency

All participants in Study 2 received an ORF assessment at the very beginning (pre-test) and end (post-test) of the study. All assessments were completed within an approximately 1-week period at pre-test and again during post-test. Each student’s WCPM and words read incorrectly per minute (WIPM) were evaluated. The passage selected for the pretest was “The Umbrella” and for the posttest the passage “The Secret of the Locker,” both passages are within the narrative genre and published within the Reading Comprehension Assessment Protocol (RCAP; Cunha and Capellini, 2014). The RCAP is appropriate for students from the third to fifth grade and the user’s manual suggests providing these two passages at pre-test and post-test like we did in Study 2. The choice to use narrative passages was because students are frequently exposed to narrative text during childhood and throughout the educational process.

##### Intervention procedures with the HELPS-PB program

The intervention was performed one-on-one (adult-student) in spaces provided by the school coordinator. The implementation period of HELPS-PB was 2 months and 25 days, beginning in August and ending in November. The HELPS-PB program was implemented approximately three times per week (every Monday, Wednesday, and Friday) for 12–14 min per session. All students received 30 intervention sessions.

The procedures of the HELPS-PB program are the same as those in the originally developed HELPS program in English (Begeny, 2009) and the subsequent Spanish version (Begeny, 2012). As a brief summary, implementation procedures include each of the evidence-based instructional and motivational strategies that past research (e.g., Therrien, 2004; Morgan and Sideridis, 2006; Lee and Yoon, 2017; Stevens et al., 2017) has found to improve students’ fluency: repeated timed readings of ability-appropriate text, modeling, phrase-drill error correction, verbal cues for the student to read with fluency and comprehension, goal setting, feedback about the student’s performance, and a structured motivational reward system {see Begeny (2009) [English] or Begeny et al. (2018a) [Portuguese] for details}. Additionally, HELPS implementation (regardless of language) incorporates 31 quality characteristics that help to ensure the most effective use of the core procedures (see instructor’s manual for details).

**Figure 2:**
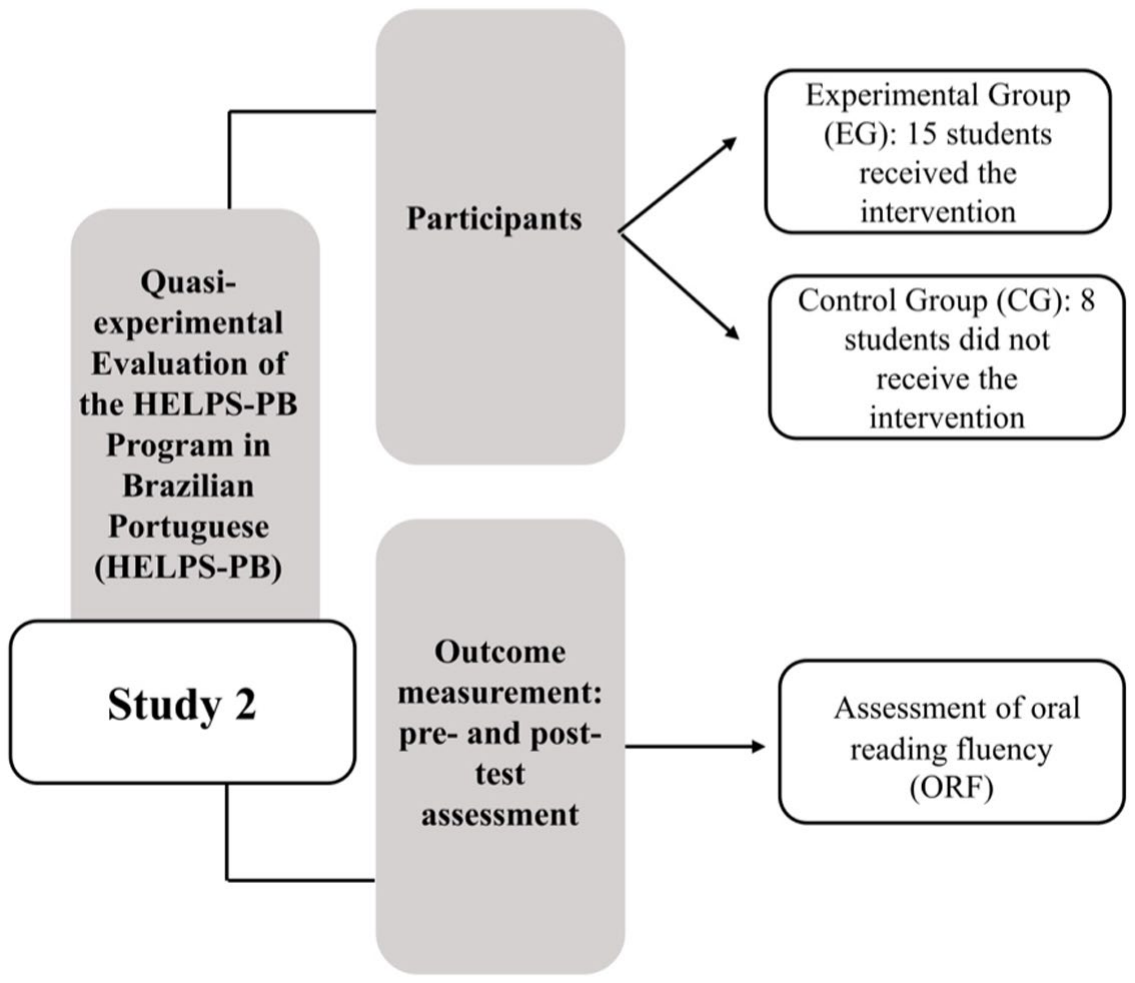
Diagram for the primary stages and activities of Study 2.

To find the ideal passage for each student to begin the HELPS-PB intervention, we followed the HELPS-PB Placement Assessment that was summarized in Study 1. In each intervention session, the instructor had the implementation flowchart and specified student directions, which were followed in each HELPS-PB session. Figure 2 presents a visual depiction of the primary stages and activities of Study 2.

##### Intervention fidelity and training

HELPS-PB was implemented by the lead researcher. At the time of the study, she had almost 10 years of experience implementing intervention programs for children with special educational needs. The Method section of Study 1 describes the extensive training this interventionist received prior to beginning Study 1.

In addition to comprehensive training in HELPS-PB, intervention fidelity was recorded at the end of each HELPS-PB session since this is a required procedure within the HELPS-PB program. That is, the final step of program requires that the instructor systematically reviews each step of the implementation sequence (i.e., of the 13 steps summarized on the one-page implementation flowchart) and then records the fidelity on the progress sheet if any of the steps were forgotten or implemented incorrectly. A previous study with this methodology demonstrated that self-recording is a reliable and valid method of assessing the implementation integrity of HELPS program procedures (Begeny et al., 2013). Thus, based on data from each student’s progress sheet, 99% of the total sessions were conducted with 100% fidelity of the core HELPS-PB procedures. That is, all 13 core steps were completed correctly.

##### Statistical analyses

To analyze possible differences in reading between the students in the experimental and control group, Mann–Whitney *U* tests (two-tailed) were used to examine possible difference in students’ WCPM and WIPM scores. This non-parametric test was the most appropriate analysis because of our relatively small sample size and because the Mann–Whitney *U* is designed for two independent (versus dependent) samples. Prior to analyses, we set our *value of p* at 0.05., and to perform these analyses, we used the *Statistical Package for Social Sciences* (SPSS-version 28.0) software.

### Study 2 results

Table 2 summarizes the average WCPM (words read correctly per minute) and WIPM (words read incorrectly per minute) scores at pre-and post-test for both the experimental and wait-list control group. As shown, students in the experimental group had, on average, somewhat lower scores at pre-test, but this difference was not statistically significant (Mann–Whitney *U* = 48.0, *n*_1_ = 15; *n*_2_ = 8, *p* = 0.44, two-tailed). Also, as stated previously, all participants were in the qualifying range for being able to benefit from a text-reading fluency intervention based on their pre-test score that was below average and generally within approximately the 30th-40th percentile based on ORF norms published by Martins and Capellini (2021).

**Table 2:** Mean, standard deviation, and range WCPM and WIPM scores at pre-and post-test by group.

Group	*n*	Score	WCPM at pre-test	WCPM at post-test	Change in WCPM^1^	WIPM at pre-test	WIPM at post-test	Change in WIPM^1^
Experimental	15	Mean	83.7	93.5	9.8^a^	3.1	2.7	−0.5
		SD	(15.2)	(13.3)	(7.4)	(2.3)	(1.5)	(2.6)
		Range	61–106	65–115	3–28	0–7	1–6	−6–4
Wait-list control	8	Mean	90.8	93.0	2.3	4.0	4.1	0.1
		SD	(11.9)	(13.8)	(7.1)	(2.5)	(3.3)	(2.4)
		Range	73–106	70–108	−9–10	1–8	0–9	−4–4

^1^Change score refers to the change between pre-and post-test. ^a^Denotes a statistically significant difference between the groups. WCPM, words read correctly per minute. WIPM, words read incorrectly per minute. SD, standard deviation (with scores shown in parentheses). Score ranges reflect students in third, fourth, and fifth grades, which helps to explain wider ranges for WCPM. Negative values in the last column reflect an improvement in reading during the post-test. The Experimental Group received HELPS-PB between the pre-and post-test period. Whereas the wait-list control group did not.

At post-test, the experimental group increased nearly 10 WCPM whereas the control group made very little improvement over time (with a mean increase of only 2.3 WCPM). This difference in improvement (i.e., the gains made by those in the intervention group compared to gains made by those in the control group) was statistically significant (Mann–Whitney *U* = 29.5, *n*_1_ = 15; *n*_2_ = 8, *p* < 0.05, two-tailed). Using an effect size calculation for the Mann–Whitney *U* test,^2^ this revealed found a large effect size: Cohen’s *d* = 0.9; Eta squared (*n^2^*) = 0.17.

With WIPM, the average score for the students in the experimental group improved somewhat between pre-and post-test whereas the average WIPM for students in the control group actually increased slightly during post-test. However, the differences were not statistically significant and, in general, both groups stayed relatively similar in their average WIPM between pre-and post-test.

Finally, although it was not a specific research question for Study 2, it is useful to note that some data from Study 2 helped to substantiate the passage sequencing and program adaptation process in Study 1. For example, the 15 participants in the experimental group had varied Placement Assessment scores, which is what would be expected if the Placement Assessment was developed well and the curriculum of passages were logically sequenced. Specifically, five students began on Passage 5; three began on Passage 25; two began on Passage 50; four began on Passage 66; and one began on Passage 75. Further, after identifying each student’s proper starting point from the Placement Assessment, successful passage development and sequencing would allow students to improve their reading in each session and ultimately meet the Reading Goal fairly regularly (e.g., usually within 1–3 sessions of practicing that passage). Data from Study 2 showed that 100% of the students who received HELPS-PB regularly met the Reading Goal in ways we would hope; thus, all 15 students practiced several new passages during the 30 sessions they received in Study 2. Across all students, with a possible maximum of practicing 29 different passages within the 30 total sessions received, the median number of passages practiced was 24 (range = 14–29). These patterns of data are highly consistent with known data for HELPS when implemented in English (e.g., Begeny et al., in press).

## Discussion of Study 1 and Study 2

National data in Brazil (e.g., OECD, 2019a; Brasil, 2021) and other countries (e.g., OECD, 2019b; National Center for Education Statistics, 2022) make it clear that millions of individuals have not yet established foundational literacy skills—which many argue is a violation of both human rights and equitable pathways to economic and quality-of-life opportunities. Reading fluency is a foundational literacy skill in most, if not all, alphabetic language systems, and evidence-based intervention programs targeting students’ text reading fluency have the capacity to improve literacy development for millions of students (e.g., Therrien, 2004; Hudson et al., 2020). Cross-cultural collaboration and possible adaptation of existing literacy programs—especially when using values and processes of internationalization in education and psychology—offer a potentially promising approach to effectively and efficiently developing high-impact instructional programs that can meet global literacy needs (Arfken, 2012; Begeny et al., 2021).

This two-part study was designed to (a) systematically translate, culturally adapt, and pilot test an existing evidence-based reading fluency program (Begeny, 2009) in order to create a version of that program (i.e., HELPS-PB) that can be used to support students learning to read in Brazilian Portuguese; and then (b) conduct an initial evaluation of HELPS-PB by using a randomized control-group quasi-experimental design. In Study 1, the systematic processes that were used (e.g., translation and back-translation, cultural adaptation, data-based curriculum sequencing, pilot implementation) ultimately led to the successful development of HELPS-PB materials and procedures. Given the systems and rigor employed in this process, it was not totally surprising to achieve successful development of HELPS-PB, as this outcome is consistent with similarly rigorous work that is often designed to translate and/or adapt other materials (often for assessment purposes) relevant to psychology and/or education (e.g., Cassepp-Borges et al., 2010; Almeida et al., 2013; Pasqualini et al., 2014). However, one should not assume such processes will lead to successful development of an adapted program, and Study 1 offers a comprehensive “blueprint” for how to achieve this with a structured literacy program such as HELPS.

Study 1 also revealed some interesting findings during the process. For instance, the translation and adaptation process led to retaining all passages adapted from the Leamos para Avanzar curriculum (Begeny et al., 2012a) and retaining several “borrowed words” from those passages. Considering that the translation of the curriculum passages sought to make the stories appropriate for most students living in Brazil, our most optimal translation and adaptation process—which sought high fidelity to the context and semantic meanings of words—did not benefit as much from following a process commonly described in the literature as word-for-word translation, which according to Barbosa (2004) reflects a literal translation. Our translation process considered the morphosyntactic changes necessary to produce the most acceptable passage in Brazilian Portuguese. This included attention to textual comprehension (Barbosa, 2004) and relevance for most Brazilian readers, while simultaneously wanting to minimize deviations from the original passage. Overall, due to the type of translation performed and its purpose, we found that there was no need to perform back translation of the HELPS curriculum passages, as this step was mainly necessary only for the HELPS instructor’s manual. This approach was also appropriate because, after the adaptation process, we gathered and analyzed data from the HELPS-PB passages to systematically sequence the passages in order of difficulty and we then developed an updated Placement Assessment and appropriate grade-level goals for HELPS-PB implementation. From this, we then validated the work even further by using pilot implementation procedures.

In Study 2 (our quasi-experimental evaluation of HELPS-PB), we found that the students randomly assigned to the experimental group significantly outperformed students in the control group on WCPM, which is the measure of reading fluency that is considered by most fluency researchers to be one of the most important, studied, and valid measures of fluency (e.g., Hasbrouck and Tindal, 2006; Lee and Yoon, 2017). Furthermore, the difference between the groups on the change/improvement of WCPM from pre-to post-test resulted in a large effect size (*d* = 0.9). This finding is very important as a promising indicator of efficacy for the newly developed HELPS-PB program. The finding is consistent with past studies on HELPS in English and Spanish (e.g., Begeny et al., 2010, 2012b; Begeny, 2011, 2019; Malouf et al., 2014) and consistent with other empirical and theoretical work in text reading fluency (e.g., Laberge and Samuels, 1974; Therrien, 2004; Stevens et al., 2017; Hudson et al., 2020). Despite this empirical consistency, this is an important preliminary finding for HELPS-PB because (a) any newly adapted intervention program should be directly evaluated for effectiveness; and (b) to our knowledge, HELPS-PB is the first and only widely available program specifically designed to target and improve text reading fluency in Brazilian Portuguese.

In Study 2 we also found that students in the experimental group lowered their WIPM from pre-to post-test, whereas students in the control group somewhat increased in WIPM. Although this is a positive direction of reducing WIPM for students who received HELPS-PB, the difference between groups was not large or statistically significant. However, this finding is not necessarily surprising because students needing support with reading fluency usually have generally good accuracy (i.e., not a lot of WIPM). Thus, with (a) a low average WIPM score to begin with (e.g., average of 3 WIPM); (b) a low opportunity for variance among the groups and thus a fairly “restricted range” in scores (e.g., 0–3); and (c) a relatively small sample size in the study—we would not expect to see statistically significant differences between the groups on WIPM even if there was some relative improvement for those receiving HELPS-PB. In this study, we felt it would be relevant to at least report the WIPM data, but we also note that in many fluency intervention studies, WIPM does not even get reported or analyzed for the reasons above (e.g., Begeny et al., 2010, 2011; Mitchell and Begeny, 2014).

### Implications

We believe our studies reported in this paper have meaningful implications for research, practice, and adapting reading fluency programs into other languages. Examples of such implications are as follows. First, Study 1 should assist reading researchers with understanding some key concepts and steps necessary in developing materials and research protocols that are essential for translating and adapting a reading intervention program in a comprehensive way and thereby preparing it to ultimately be evaluated in an experimental or quasi-experimental study (or optimally, a series of studies). We also encourage such researchers to consider adaptation and collaboration based on values and processes associated with internationalization (Arfken, 2012; van de Vijver, 2013; Begeny, 2018a), which should help to avoid the all-too-common “over-Westernization” of programming in non-Western contexts where that may be harmful (see, for example, Bernardo et al., 2018; Begeny et al., 2021). Indeed, a great deal of past work has used cross-cultural translations and adaptations, particularly when considering methods of assessment in psychology and/or education (van de Vijver, 2013); but much less appears to be written about comprehensive cross-cultural adaptation of targeted academic interventions that may have applicability to improve students’ learning on a global scale. Thus, the present report offers one example that researchers can consider in this regard.

Another implication of our work and dissemination model for HELPS-PB allows easier opportunity for interested researchers to conduct additional efficacy or effectiveness studies on HELPS-PB—which is greatly needed at this time because multiple studies are needed to strengthen confidence and understanding about a program’s impact. With HELPS-PB now fully developed and freely available for download, along with Study 2 showing initial indicators of efficacy for HELPS-PB, we encourage interested researchers to continue evaluating the impact of this program so that there is greater understanding about the contexts where it is effective and the variables (e.g., student grade level, type of interventionist) that may influence effectiveness. This model of dissemination was used with the original HELPS program and, to date, has assisted in better understanding the impact of HELPS in a range of different contexts.

In terms of examples of implications for practice, we first highlight that, as result of our studies, educators in Brazil now have an intervention program (HELPS-PB) to use with the many students in Brazil who struggle with reading fluency. Again, the free access to HELPS-PB, including all implementation materials and the do-it-yourself training materials (including freely accessible video demonstrations), may make this program appealing to teachers who need to better support students’ reading fluency and are looking for a program to accomplish that. Indeed, just by having the program available online, the initial year has already resulted in hundreds of downloads by Brazilian educators. Additionally, our team, in collaboration with Helps Education Fund, aims to make video-based (e.g., with Zoom) or in-person training for HELPS-PB free or low cost for Brazilian educators who prefer that approach to training over the do-it-yourself model.

With this, we recognize the time-based limitation of using a one-on-one (adult-student) intervention program, but there are numerous ways in which the versatility of this intervention has made it widely usable and feasible for educators at the classroom, school, or district level (Begeny, 2009; Begeny et al., in press). Examples of what can make HELPS-PB versatile include (a) options to train non-education experts (e.g., community volunteers or university students preparing to be an educator) to implement the program with fidelity; (b) the feasible “dosage” needed for effectiveness (e.g., 15 min per session, three times per week); and (c) the ability for the program to be easily used by multiple interventionists with the same student. Also, a small-group version of the program can be implemented with multiple students at once, and our team is already in the process of translating the HELPS-SG instructor’s manual (Begeny, 2018b) so that educators in Brazil have the additional option of using HELPS-PB with multiple students at once. Fortunately, the same HELPS-PB curriculum of passages developed in Study 1 is the curriculum needed for a forthcoming small-group version of HELPS-PB.

Finally, we believe another implication of this report is that Study 1 and 2 provide a relatively clear blueprint for other researchers, educators, or education administrators to consider if they are specifically interested in adapting the HELPS intervention program into other languages—in addition to what is currently available in English, Spanish, and Brazilian Portuguese. Such work may allow greater opportunity to support students around the globe who have not yet developed proficient text reading fluency in their native language.

### Limitations and directions for future research

Both of our studies reported in this paper are not without limitations. For example, Study 1 ultimately allowed for an appropriate data collection process for purposes of sequencing the curriculum and developing implementation tools (i.e., the Placement Assessment) and guidelines (i.e., the Reading Goal levels for Brazilian Portuguese). However, this process could have been strengthened if it had included students in grades 2–4 and involved a larger number of students per grade level who could all read the initial set of HELPS-PB passages. For example, including 30–50 students across students in grade 2, 3, and 4—all of whom share similar grade-level oral reading fluency—would strengthen the process. Fortunately, the pilot implementation (Study 1) and subsequent quasi-experimental study helped to validate decisions on curriculum sequencing; but this limitation should still be considered as efficacy and effectiveness studies continue over time with HELPS-PB.

Pilot implementation and experimental work could also be improved in future research. For example, researchers are encouraged to collect acceptability and usability data (collected systematically through surveys and/or interviews) from (a) students who receive HELPS-PB; (b) interventionists who deliver it; and (c) classroom teachers of students who receive the program, if those teachers do not serve as the interventionist. Similar to current research with HELPS in English and Spanish, future research with HELPS-PB should also include different types of interventionists (e.g., classroom teachers, other school staff, teachers in training, community volunteers, etc.). Past studies with HELPS show that all types of interventionists can be equally effective as long as they receive proper training and implement with fidelity, but this type of research specifically with HELPS-PB would be beneficial.

Furthermore, future studies of HELPS-PB would benefit from including larger sample sizes for experimental and control groups, as well as using additional measures of student performance (e.g., multiple measures of oral reading fluency; robust measures of reading comprehension and/or prosody that are psychometrically supported and do not restrict variability in scored performance). These types of directions for future research are common for nearly all intervention research (especially newly developed programs), so we readily acknowledge that such work will greatly enhance the existing research-base of HELPS-PB. Such research with larger samples will also minimize Type 2 error. For example, in Study 2, the very small sample size significantly increased the probability of not finding a statistically significant effect. The fact that we still observed a statistically significant difference between the groups in students’ improvement of WCPM suggests there was a strong and promising effect; but future studies should aim to minimize Type 2 error by having a sample size that supports a beta level of 0.20 or lower (i.e., having statistical power at 0.80 or higher).

Additional research should also systematically evaluate training and coaching procedures for educators and other interventionists who want to use HELPS-PB in their educational contexts. Such research will complement usability and acceptability studies of the HELPS-PB program by specifically examining the variables, challenges, and successes that come with training and coaching interventionists to use HELPS-PB in a variety of educational contexts. Similarly, future research should consider completing and reporting even more comprehensive evaluations of intervention fidelity. The present study monitored and reported fidelity in one way that has been supported by past HELPS research (e.g., Begeny et al., 2013), but most of the past research with HELPS also includes a report of intervention fidelity as determined by an independent observer who documents the fidelity of at least 20–35% of each interventionist’s HELPS sessions with students—and we encourage this added level of fidelity reporting in future studies.

## Conclusion

This report summarizes a 5-year project that ultimately achieved a fully adapted version of an evidence-based reading fluency intervention into Brazilian Portuguese and the first quasi-experimental evaluation of the program. HELPS-PB is now available for researchers and educators to potentially use and/or further research, and such work will hopefully allow for an expanded knowledge-base for HELPS-PB usability and effectiveness. HELPS-PB is simply one potentially promising tool to assist the millions of students in Brazil who have not developed proficient reading skills; but such a program and/or its iterations that may come from additional research and development, offers promise for providing students with a more equitable and effective learning experience—one that results in proficient reading skills and the opportunities that come from being a confident, proficient reader.

## Data availability statement

The original contributions presented in the study are included in the article/supplementary material, further inquiries can be directed to the corresponding author.

## Ethics statement

The studies involving human participants were reviewed and approved by UNESP—Faculdade de Filosofia e Ciências—Campus de Marília. Written informed consent to participate in this study was provided by the participants’ legal guardian/next of kin.

## Author contributions

MM designed and executed the study, performed the data collection, and wrote the manuscript and final revision of the manuscript. JB and SC designed the study, assisted with the data analyses, critically reviewed the theoretical content, and revised the manuscript. All authors have read and approved the final manuscript.

## Funding

Results of this publication involved funding for MM, which was provided by the National Council for Scientific and Technological Development-CNPq - process no 140304/2014-2, with a funding period from March 2014 to February 2018. The funding was also provided for MM to complete a PhD.

## Conflict of interest

The authors declare that the research was conducted in the absence of any commercial or financial relationships that could be construed as a potential conflict of interest.

## Publisher’s note

All claims expressed in this article are solely those of the authors and do not necessarily represent those of their affiliated organizations, or those of the publisher, the editors and the reviewers. Any product that may be evaluated in this article, or claim that may be made by its manufacturer, is not guaranteed or endorsed by the publisher.

## Footnotes

^1^Available at: http://michaelis.uol.com.br/moderno-portugues/

^2^ https://www.psychometrica.de/effect_size.html
